# Serum lipids, retinoic acid and phenol red differentially regulate expression of keratins K1, K10 and K2 in cultured keratinocytes

**DOI:** 10.1038/s41598-020-61640-9

**Published:** 2020-03-16

**Authors:** Hebah Aldehlawi, Saima Usman, Anand Lalli, Fatima Ahmad, Gianne Williams, Muy-Teck Teh, Ahmad Waseem

**Affiliations:** 1 College of Dentistry, King Abdulaziz University, Jeddah, Kingdom of Saudi Arabia; 2 Centre for Oral Immunobiology and Regenerative Medicine, Institute of Dentistry, Barts & The London School of Medicine and Dentistry, Queen Mary University of London, England, United Kingdom; 3 China-British Joint Molecular Head and Neck Cancer Research Laboratory, Affiliated Stomatological Hospital of Guizhou Medical University, Guizhou, China; 4 Cancer Research Institute, Affiliated Cancer Hospital and Institute of Guangzhou Medical University, Guangzhou, China

**Keywords:** Cancer, Cell biology, Molecular biology, Biomarkers

## Abstract

Abnormal keratinocyte differentiation is fundamental to pathologies such as skin cancer and mucosal inflammatory diseases. The ability to grow keratinocytes *in vitro* allows the study of differentiation however any translational value is limited if keratinocytes get altered by the culture method. Although serum lipids (SLPs) and phenol red (PR) are ubiquitous components of culture media their effect on differentiation is largely unknown. We show for the first time that PR and SLP themselves suppress expression of differentiation-specific keratins K1, K10 and K2 in normal human epidermal keratinocytes (NHEK) and two important cell lines, HaCaT and N/TERT-1. Removal of SLP increased expression of K1, K10 and K2 in 2D and 3D cultures, which was further enhanced in the absence of PR. The effect was reversed for K1 and K10 by adding all-trans retinoic acid (ATRA) but increased for K2 in the absence of PR. Furthermore, retinoid regulation of differentiation-specific keratins involves post-transcriptional mechanisms as we show *KRT2* mRNA is stabilised whilst *KRT1* and *KRT10* mRNAs are destabilised in the presence of ATRA. Taken together, our results indicate that the presence of PR and SLP in cell culture media may significantly impact *in vitro* studies of keratinocyte differentiation.

## Introduction

Skin is the main body barrier against environmental stresses including UV, chemicals and microbes as well as providing protection against water loss^1,2^. It has two compartments, the uppermost epidermis which directly faces different environmental insults, and the underlying dermis, which is rich in connective tissue including blood vessels, nerves and fibroblasts to support the epidermis and runs the biological functions of the skin^3^. The epidermis is made up of keratinocytes, arranged in a multi-layered fashion in which the deepest basal layer contains mitotically active cells, a small proportion of these cells are stem cells^4,5^, which are responsible for all repair and maintenances of the epidermis^6,7^. The supra-basal layers overlying the basal layer contains mostly differentiating keratinocytes. The basal keratinocytes divide and as the progeny move upward they embark on a programme of differentiation associated with the expression of keratin genes^8^. Studying the regulation of keratinocyte differentiation is fundamental to understanding many skin and mucosal pathologies such as cancer, inflammatory diseases as well as wound healing. This relies on the ability to grow keratinocytes *in vitro* which have similar characteristics to those found in nature. If culture conditions were to significantly influence keratinocyte differentiation biomarkers, this could lead to misinterpretation of clinical significance and limit the translational value of these laboratory studies.

About 80% of all proteins in keratinocytes are keratins, a very large family of structural proteins, which assemble first into heterocomplexes of type I and type II keratins in a ratio of 1:1 and then into intermediate filaments^9,10^. In epidermis, the keratin expression is tightly regulated with different layers expressing intermediate filaments made of different type I/type II keratin pairs. The basal keratinocytes mainly express K5/K14^11–13^ with K15 as a minor component^14,15^. When basal keratinocytes move into the *stratum spinosum* they downregulate K5/K14/K15 and begin to express differentiation-specific keratins K1/K10^16–18^. As the differentiating keratinocytes move further up into the *stratum granulosum* another type II keratin, K2, which also pairs with K10 is induced^19,20^. This differentiation programme produces several cell layers containing keratinocytes at different stages of differentiation until the cells are terminally differentiated before shedding off from the skin surface. In hyperproliferative situations such as during wound healing, psoriasis, pathological scarring and in some cancers, keratins K6, K16 and K17 are induced^20–23^. The terminally differentiated keratinocytes in epidermis also express a number of other differentiation markers including involucrin^24,25^, filaggrin^26^, loricrin^27,28^, cornifins^29^ and transglutaminases^30^.

Differentiation of keratinocytes in skin is regulated by a large number of environmental and dietary factors including retinoids, a group of vitamin A derivatives, which target specifically the epidermal layers of the skin^31–33^. Earlier studies have suggested that retinoids suppress the terminal differentiation of keratinocytes including formation of cornified envelopes^34,35^ and suppression of terminal differentiation markers loricin^36^, cornifin^37^, and transglutaminase I^38^. Retinoids also suppress expression of a number of keratins including K1, K10, K5, K14, K6, K16^34,39–42^ in cultured keratinocytes. The retinoid induced inhibition of expression of keratins has been shown to involve transcriptional suppression mediated by direct interaction between ligand occupied nuclear receptors and retinoid-responsive elements present on keratin genes^42–45^. In addition to transcriptional regulation, post-transcriptional regulation of *KRT19* has also been reported^43^.

Most studies described in the literature have used lipid free serum to evaluate the influence of effectors such as retinoids or steroids on keratin gene expression. In this manuscript, we have studied the effect of charcoal-stripped foetal calf serum (CS-FCS) on the expression of differentiation-specific keratins, K1, K10 and K2 in normal human epidermal keratinocytes (NHEK), HaCaT and N/TERT-1 cells and compared it with unstripped FCS. We show that expression of *KRT1*, *KRT10* and *KRT2* is increased in CS-FCS but suppressed by serum lipids (SLP) and phenol red (PR). The effect of PR on keratinocyte differentiation is novel and it is consistent with previous reports where PR has been shown to have a protective effect against solar simulated radiation^46^. We show that some effects of SLP could be mimicked by all-trans-retinoic acid (ATRA) which suppressed the expression of *KRT1* and *KRT10* but increased *KRT2* expression. We demonstrate that the increase in *KRT2* transcript was because of stabilisation of its mRNA by ATRA whereas *KRT1* and *KRT10* mRNAs were destabilised. This is the first report where differential effect of retinoids on the expression of differentiation-specific keratins has been ascribed to the stability of their transcripts.

## Results

### Presence of SLPs and PR suppresses expression of differentiation-specific keratins in NHEK

To investigate whether lipids present in FCS and PR present in culture medium would influence expression of differentiation-specific keratin genes *KRT1*, *KRT10* and *KRT2* in human keratinocytes, we used SLP deficient CS-FCS and PR free RM^+^ culture medium. In our experiments, we have grown NHEK in four different culture conditions (SLP^+^/PR^+^, SLP^−^/PR^+^, SLP^+^/PR^−^, SLP^−^/PR^−^) for three days before evaluating specific mRNA levels by qPCR and protein expression by western blotting.

**Figure 1:**
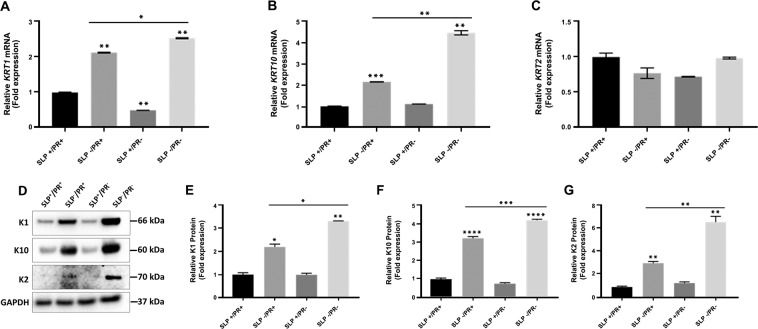
Effect of serum lipids (SLP) and phenol red (PR) on the expression of *KRT1*, *KRT10* and *KRT2* in NHEK cells. NHEK (200,000 cells) were mixed with 10^6^ irradiated 3T3 fibroblasts in 2 ml growth medium per well of a 12 well plate for 3 days in four different RM^+^ media conditions, PR containing RM^+^ with or without SLP containing FCS (SLP^+^/PR^+^, SLP^−^/PR^+^), no PR in RM^+^ with or without SLP containing FCS (SLP^+^/PR^−^, SLP^−^/PR^−^) in triplicates for each growth condition. Feeder cells were removed by squirting PBS/EDTA and keratinocyte lysates were collected and analysed using qPCR and WB. mRNA expression data are shown for different genes (**A**) *KRT1*, (**B**) *KRT10* and (**C**) *KRT2* as fold expression normalised to the expression of two housekeeping genes, *POLR2A* and *YAP1* under different growth conditions. (**D**) NHEK (500,000 cells) were mixed with 1 million irradiated 3T3 fibroblasts in 4 ml growth medium per well of a 6 well plate for 3 days in the above four different RM^+^ media conditions in triplicates for each growth condition. Feeders were removed as above and SDS cell lysate containing 20 µg total protein (measured by Lowry’s method using BSA as standard) was analysed by western blotting for expression of K1, K2 and K10 under different growth conditions. GAPDH was used as a loading control. Relevant bands were cropped from different blots and grouped together. Original blots are shown in Supplementary Fig. S1. Quantification of protein bands was carried out by Image J and shown for (**E**) K1, (**F**) K10 and (**G**) K2. Statistical analyses: n = 3, Error bars = SEM, Student’s t-test was performed to calculate p values using Microsoft Excel. One-way ANOVA (shown by a horizontal line over the graph) was used to determine the statistical significance in gene expression between different growth conditions. The p values are given by asterisks (*p < 0.05, **p < 0.01, ***p < 0.001 and ****p < 0.0001).

As shown in Fig. 1A,B we observed upregulation of *KRT1* and *KRT10* mRNA expression in CS-FCS containing medium with PR (SLP^−^/PR^+^) compared with SLP^+^/PR^+^ medium suggesting that the presence of SLPs suppress *KRT1*/*KRT10* expression. The expression of *KRT1* and *KRT10* was further enhanced in the absence of PR (SLP^−^/PR^−^) suggesting PR also suppresses *KRT1* and *KRT10* expression in the absence of SLP (compare SLP^−^/PR^**+**^ with SLP^−^/PR^−^ in Fig. 1A,B, Table 1). In the presence of SLPs, however, PR appears to increase only *KRT1* but not *KRT10* expression (Fig. 1A,B, Table 1). The *KRT10* gene can produce two spliced variants, *KRT10* and *KRT10X1* transcripts, translating into polypeptides of 584 and 624 residues, respectively^47^. It should be noted that the primer set we have used in this study will not be able to differentiate between the two transcripts. Interestingly, *KRT2* mRNA which is also expressed in the suprabasal epidermal keratinocytes didn’t show any significant difference in the presence or absence of SLP and/or PR (Fig. 1C, Table 1). The protein expression truly mimicked the mRNA expression for *KRT1* and *KRT10* with much higher protein expression in SLP^−^/PR^+^ compared with the control SLP^+^/PR^+^ but did not change significantly in SLP^+^/PR^−^. Removing the SLP and PR (SLP^−^/PR^−^) from the medium synergistically increased K1 and K10 expression (Fig. 1D–F). A similar pattern was also observed for K2 expression although the levels expressed were very low requiring much longer exposure to detect the protein band (Fig. 1D,G).

**Table 1:** mRNA expression of *KRT1*, *KRT10* and *KRT2* in four different culture conditions in NHEK, HaCaT and N/TERT-1 cells.

Cell types	Genes	Fold expression ± SEM			
		SLP^+^/PR^+^*	SLP^-^/PR^+^*	SLP^+^/PR^−^*	SLP^-^/PR^−^*
NHEK	*KRT1*	1 ± 0.02	2.13 ± 0.02	0.49 ± 0.003	2.54 ± 0.02
	*KRT10*	1 ± 0.01	2.15 ± 0.009	1.1 ± 0.002	4.46 ± 0.10
	*KRT2*	1 ± 0.05	0.77 ± 0.07	0.72 ± 0.005	0.98 ± 0.01
HaCaT	*KRT1*	1 ± 0.08	2.4 ± 0.16	0.8 ± 0.106	2.5 ± 0.41
	*KRT10*	1 ± 0.11	26.2 ± 1.97	15.6 ± 2.33	38.4 ± 5.81
	*KRT2*	1 ± 0.057	0.97 ± 0.133	1.3 ± 0.131	0.86 ± 0.07
N/TERT-1	*KRT1*	1 ± 0.10	0.92 ± 0.14	3.49 ± 0.12	1.42 ± 0.15
	*KRT10*	1 ± 0.02	0.76 ± 0.06	2.71 ± 0.28	1.18 ± 0.06
	*KRT2*	1 ± 0.02	0.83 ± 0.02	1.11 ± 0.02	0.98 ± 0.08

*SLP^+^/PR^+^: complete FCS with phenol red (PR); SLP^−^/PR^+^: charcoal stripped FCS (CS-FCS) with PR; SL^+^/PR^−^: complete FCS without PR; SLP^−^/PR^−^: CS-FCS without PR. Each experiment was performed at least in 3 independent replicates. Standard error of the mean (SEM) was calculated using Microsoft Excel.

### Effect of SLP and PR on the expression of differentiation-specific keratins in 2D and 3D cultures

**Figure 2:**
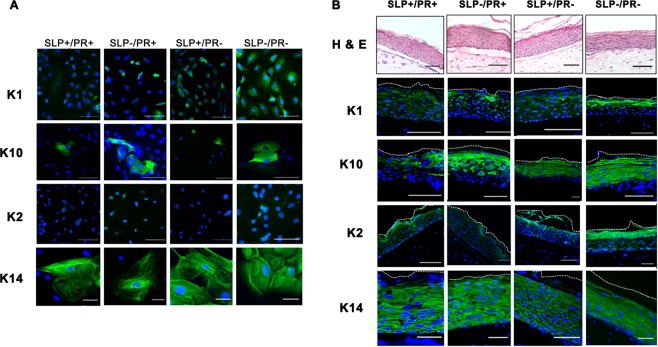
Effect of SLP and PR on the expression of K1, K10 and K2 in 2D and 3D cultures. (**A**) NHEK (50,000 cells) were grown with 100,000 irradiated 3T3 feeder cells on glass coverslips in 50 µl medium and after the cells had attached, 1 ml of four different RM^+^ media conditions, PR containing RM^+^ with or without SLP containing FCS (SLP^+^/PR^+^, SLP^−^/PR^+^), no PR in RM^+^ with or without SLP containing FCS (SLP^+^/PR^−^, SLP^−^/PR^−^) was added. The cells on coverslips were immunostained for K1, K10, K2 and K14. (**B**) NHEK (500,000 cells) were grown in 3D cultures in an insert (pore size 0.4 µm) based method. Primary dermal fibroblasts (100,000 cells/insert) were used in 400 µl collagen matrix to support the growth of NHEK. The cells were grown at air-liquid interface for 10 days with 4 different media conditions (**A**: SLP^+^/PR^+^, **B**: SLP^−^/PR^+^, **C**: SLP^+^/PR^−^ and **D**: SLP^−^/PR^−^), fixed in 4% (w/v) paraformaldehyde/PBS and paraffin embedded. Sections of 5 µm thickness were cut, de-waxed, re-hydrated, H & E stained, de-hydrated and mounted using DPX medium. Nikon Eclipse 80i Stereology Microscope was used for recording. (Scale bar =100 µm). To immunostain the 3D cultures, the re-hydrated sections were antigen retrieved, followed by immunostaining with antibodies against K1, K10, K2 and K14, the nuclei were counterstained with DAPI in blue, overlapping images of DAPI with keratin staining is shown as merged images. Leica DM4000B Epi-fluorescence microscope equipped with DFC350 camera was used for recording. (scale bar = 20 μm). The upper most stratum corneum has been indicated by white dashed line.

We investigated the effect of SLP and PR on the expression of these keratins in NHEK cells growing in 2D cultures by immunocytochemistry. In monolayer cultures there was a clear difference in the K1, K10 and K2 expression when SLP and PR were removed from the medium but there was no difference in the expression of K14 (Fig. 2A).

To investigate whether the presence of SLP and PR would affect K1, K10 and K2 expression in differentiating keratinocytes, we produced 3D cultures using NHEK and human dermal fibroblasts (HDF) under the 4 different conditions of SLP and PR (SLP^+^/PR^+^, SLP^−^/PR^+^, SLP^+^/PR^−^, SLP^−^/PR^−^). Although the 3D cultures were grown for 10 days, the morphology of the suprabasal layers indicates that terminal differentiation was not complete. Nevertheless, keratinocyte differentiation was evident as indicated by expression of differentiation-specific markers. As expected, removal of SLP in the absence of PR induced keratinisation of the stratified epithelia. As shown in Fig. 2B, the lowest expression of K1 and K10 was observed in keratinocytes grown in SLP and PR containing medium (SLP^+^/PR^+^) and the highest expression was observed when SLP were removed and the medium did not contain PR (SLP^−^/PR^−^). This shows that keratinocytes became more differentiated in SLP^−^/PR^−^ medium. This set of data is consistent with the western blotting data presented in Fig. 1D–G. The expression of K2 followed a pattern similar to K1 and K10 in the four different conditions except that the level of expression was low. In the absence of SLP and PR (SLP^−^/PR^−^) expression of K2 was strongly induced in keratinising layers (Fig. 2B).

### Influence of SLP and PR on the expression of *KRT1*, *KRT10* and *KRT2* in HaCaT and N/TERT-1 cells

**Figure 3:**
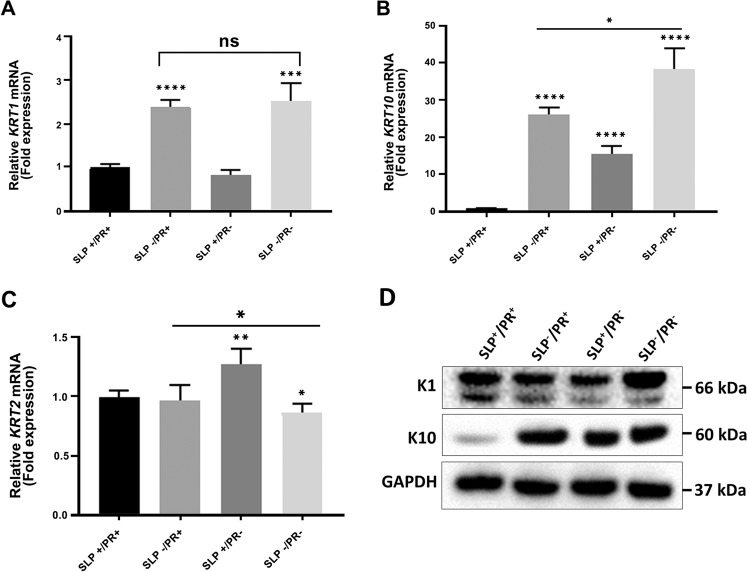
Influence of SLP and PR on mRNA and protein expression of K1, K10 and K2 in HaCaT cells. HaCaT (200,000 cells) were grown in 2 ml growth medium per well of a 12 well plate for 3 days in four different RM^+^ media conditions (SLP^+^/PR^+^, SLP^−^/PR^+^, SLP^+^/PR^−^, SLP^−^/PR^−^) in triplicates for each growth condition. Lysates were collected and analysed for mRNA using qPCR. The mRNA expression data are shown for (**A**) *KRT1*, (**B**) *KRT10* and (**C**) *KRT2* as fold expression normalised to the expression of two housekeeping genes, *POLR2A* and *YAP1* under different growth conditions. (**D**) HaCaT (500,000 cells) were grown in 4 ml growth medium per well of a 6 well plate for 3 days in the above four different RM^+^ media conditions in triplicates for each growth condition. SDS cell lysate containing 20 µg total protein (measured by Lowry’s method using BSA as standard) was analysed by western blotting for K1, and K10 under different growth conditions. GAPDH was used as a loading control. Relevant bands were cropped from different blots and grouped together. Original blots are shown in Supplementary Fig. S2. Statistical analyses: n = 3, Error bars = SEM, Student’s t-test was performed to calculate p values using Microsoft Excel. One-way ANOVA (shown by a horizontal line over each graph) was used to determine the statistical significance in gene expression between different growth conditions. The p values are given by asterisks (ns = p > 0.05, *p < 0.05, **p < 0.01, ***p < 0.001 and ****p < 0.0001).

**Figure 4:**
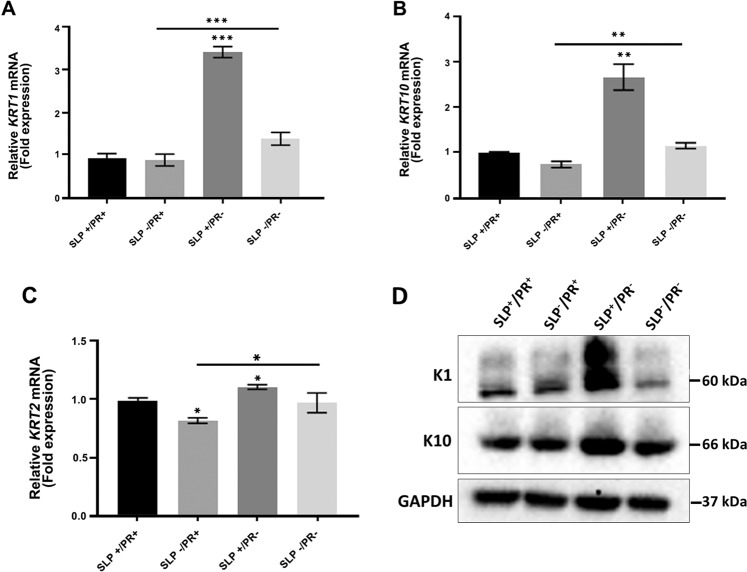
Influence of SLP and PR on mRNA and protein expression of K1, K10 and K2 in N/TERT-1 cells. N/TERT-1 (200,000 cells) were grown in 2 ml growth medium per well of a 12 well plate for 3 days in four different RM^+^ media conditions (SLP^+^/PR^+^, SLP^−^/PR^+^ SLP^+^/PR^−^, SLP^−^/PR^−^) in triplicates for each growth condition. Lysates were collected and analysed for mRNA using qPCR. The mRNA expression data are shown for (**A**) *KRT1*, (**B**) *KRT10* and (**C**) *KRT2* as fold expression normalised to the expression of two housekeeping genes, *POLR2A* and *YAP1* under different growth conditions. (**D**) N/TERT-1 (500,000 cells) were grown in 4 ml growth medium per well of a 6 well plate for 3 days in the above four different RM^+^ media conditions in triplicates for each growth condition. SDS cell lysate containing 20 µg total protein (measured by Lowry’s method using BSA as standard) was analysed by western blotting for K1 and K10 proteins under different growth conditions. GAPDH was used as a loading control. Relevant bands were cropped from different blots and grouped together. Original blots are shown in Supplementary Fig. S3. Statistical analyses: n = 3, Error bars = SEM, Student’s t-test was performed to calculate p values using Microsoft Excel. One-way ANOVA (shown by a horizontal line over each graph) was used to determine the statistical significance in gene expression between different growth conditions. The p values are given by asterisks (*p < 0.05, **p < 0.01, and ***p < 0.001).

HaCaT and N/TERT-1 are immortal keratinocyte cell lines derived from epidermis that are widely used as substitutes for NHEK^48–51^. We compared the effect of SLP and PR on *KRT1*, *KRT10* and *KRT2* expression in HaCaT and N/TERT-1 cells. Neither of these lines expressed detectable quantities of K2 under any of our experimental conditions. As shown in Fig. 3, the effect of SLP and PR on the expression of *KRT1* and *KRT10* in HaCaT was broadly similar to NHEK (Fig. 3A,B, Table 1) with the difference that *KRT10* mRNA and protein expression in SLP^+^/PR^+^ was very low (Fig. 3B,D, Table 1). Absence of SLP with PR (SLP^−^/PR^+^) did not make any difference in the level of *KRT2* mRNA expression, however, it was increased significantly in the medium containing SLP^+^/PR^−^ (Fig. 3C, Table 1). In N/TERT-1 cells, removal of SLP in the presence of PR (SLP^−^/PR^+^) did not change *KRT1* and *KRT10* expression but decreased *KRT2* expression. However, K1 and K10 expression was increased in SLP^+^/PR^−^ compared with SLP^+^/PR^+^ and SLP^−^/PR^+^ (Fig. 4D). PR was able to suppress *KRT1*, *KRT10* and *KRT2* expression in the presence of SLP but both the mRNA and protein expression increased when PR was removed from the medium (compare SLP^+^/PR^+^ with SLP^+^/PR^−^ in Fig. 4A–D, Table 1). This expression pattern is different from the one obtained with HaCaT (Fig. 3). These data suggest that the method of immortalisation also determines how these genes respond to SLP and PR.

### Influence of ATRA and PR on *KRT1*, *KRT10* and *KRT2* mRNA expression in NHEK

**Figure 5:**
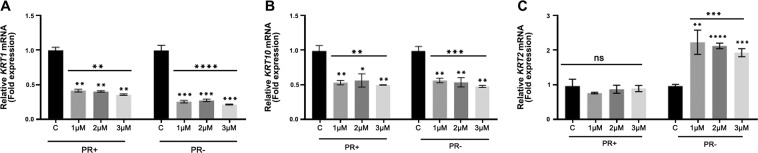
ATRA supresses *KRT1* and *KRT10* expression while *KRT2* expression is increased. NHEK (200,000 cells) were mixed with 10^6^ irradiated 3T3 fibroblasts in 2 ml of CS-FCS containing RM^+^ with or without PR per well of a 12 well plate in triplicates for each ATRA concentration for 3 days. ATRA stock was made in DMSO and further diluted in ethanol (EtOH). The cells were grown in three different ATRA concentrations of 1 µM, 2 µM and 3 µM and DMSO/EtOH was used as a vehicle control (0.003%/0.03% maximum) for 24 h after which the feeder was removed by squirting with PBS/EDTA and lysates were collected for qPCR analysis for (**A**) *KRT1*, (**B**) *KRT10* and (**C**) *KRT2*. Data are shown as fold expression normalised to the expression of two housekeeping genes, *POLR2A* and *YAP1*. Statistical analyses: n = 3, Error bars =SEM. One-way ANOVA (shown by a horizontal line over each graph) to measure the p values at different concentrations of ATRA compared to the control (no ATRA). Two-way ANOVA was used to measure the statistical significance between PR^+^ and PR^-^ groups for each keratin mRNA (*KRT1, KRT10, and KRT2*), p values are given by asterisks (ns = p > 0.05, *p < 0.05, **p < 0.01, ***p < 0.001 and ****p < 0.0001).

Although SLP include fatty acids, retinoic acids and their metabolites, we argued that the effect of removing SLP from FCS was primarily due to the presence of retinoids in SLP. We therefore attempted to replicate the effect of SLP by adding back all-trans retinoic acid (ATRA) and evaluating the expression of *KRT1*, *KRT10* and *KRT2*. NHEK were grown with γ-irradiated feeder fibroblasts in RM^+^ with CS-FCS (SLP^−^) either in the presence or absence of PR. ATRA was added at a final concentration of 1 µM, 2 µM or 3 µM in NHEK culture medium for 24 h after which the cells were lysed to estimate mRNA expression by qPCR. As shown in Fig. 5, the mRNA expression of both *KRT1* and *KRT10* was reduced significantly in the presence of ATRA whether PR was present or not. Presence of PR reduced the effect of ATRA on *KRT1* but not *KRT10* (Fig. 5A,B). For *KRT2*, the expression pattern was starkly different as in the presence of PR the *KRT2* mRNA was not influenced by ATRA but showed significant increase in the absence of PR (Fig. 5C).

**Figure 6:**
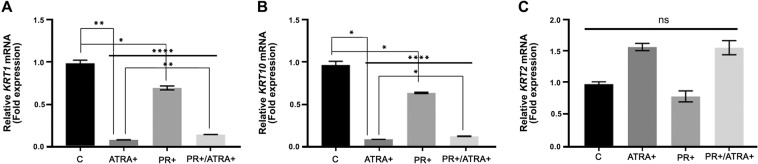
PR supresses mRNA expression for *KRT1* and *KRT10* in NHEK. NHEK (200,000 cells) were mixed with 10^6^ irradiated 3T3 fibroblasts in 2 ml of CS-FCS containing and PR free RM^+^ medium per well of a 12 well plate in triplicates for each growth condition. PR was dissolved in this medium at a concentration similar to that used in normal culture medium (0.01 mg/ml). NHEK were cultured in this medium for 3 days either with or without 1 µM ATRA added in the last 24. The feeder was removed by squirting PBS/EDTA after which cell lysates were collected for qPCR gene expression analysis for (**A**) *KRT1*, (**B**) *KRT10* and (**C**) *KRT2*. Control cells were treated with DMSO/EtOH (0.001%/0.01%). Data are shown as fold expression normalised to the expression of two housekeeping genes, *POLR2A* and *YAP1*. Statistical analyses: n = 3, Error bars =SEM, Student’s t-test was performed to calculate p-values using Microsoft Exel. One-way ANOVA (shown by a horizontal line over each graph) was used to determine the statistical significance in gene expression between different growth conditions. The p values are shown by asterisks (ns = p > 0.05, *p < 0.05, **p < 0.01 and ****p < 0.0001).

To further substantiate the effect of PR, we grew NHEK with feeder cells in SLP^−^/PR^−^ RM^+^ medium in the absence of PR. Cells were grown for 72 h with 1 µM of ATRA or 0.01 mg/ml PR (the concentration used in normal culture medium) added in the last 24 h before harvesting the cells for qPCR analyses. As shown in Fig. 6, the expression of *KRT1* and *KRT10* was reduced in ATRA as well as in PR treated cells (Fig. 6A,B). However, when PR and ATRA were added together the effect was not synergistic, instead PR reduced the effect of ATRA on *KRT1* and *KRT10* expression (Fig. 6A,B). Interestingly, the expression of *KRT2* in presence of ATRA did not change (Fig. 6C). This shows that ATRA treatment suppresses steady state level of *KRT1* and *KRT10* mRNA but does not influence *KRT2* mRNA level.

### Effect of β-Estradiol on the expression of *KRT1*, *KRT10* and *KRT2* mRNA in NHEK

Due to the reported structural similarity between PR and β-Estradiol (ED)^52,53^, we hypothesised that PR could be mimicking the effect of ED. We therefore investigated the effect of ED on *KRT1*, *KRT10* and *KRT2* expression. NHEK were grown with feeder cells in PR free RM^+^ medium with CS-FCS (SLP^−^/PR^−^) before adding ED at different concentrations for 24 h followed by cell lysis for qPCR analysis. There was no systematic change in *KRT1*, *KRT10* and *KRT2* expression, instead increasing only at certain concentrations of ED (Supplementary Fig. S4). This suggests that the effect of PR observed in this study could not be due to the structural similarity between PR and ED (Figs. 1, 3, 4 and 6).

### Differential stability of *KRT1*, *KRT10* and *KRT2* mRNA in NHEK

**Figure 7:**
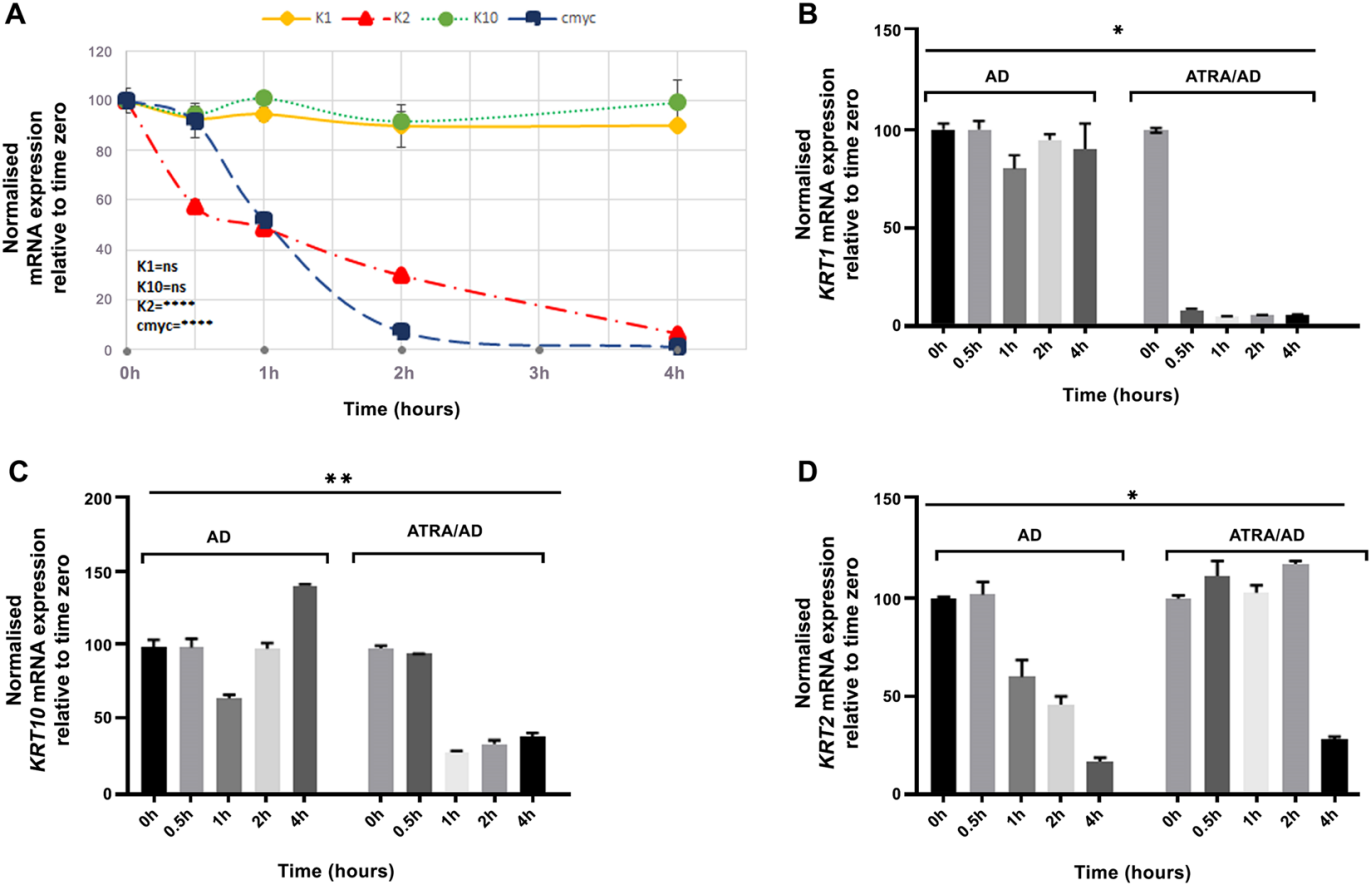
Differential stability of *KRT1*, *KRT10*, *KRT2* and *c-MYC* mRNA in NHEK. (**A**) NHEK (200,000 cells) mixed with 10^6^ irradiated 3T3 fibroblasts were co-cultured in 2 ml RM^+^ medium containing PR per well of a 12 well plate in triplicates for each gene. Cells were treated with 2 µg/ml AD for up to 4 h, the feeder was removed by squirting PBS/EDTA and the NHEK were lysed for qPCR analysis of *KRT1* (yellow diamond), *KRT10* (green circle), *KRT2* (red triangle) and *c-MYC* (blue square) gene expression analyses. All data are shown as relative expression normalised to *POLR2A* and *YAP1*, untreated cells were used as zero-time control. NHEK (200,000 cells) mixed with 10^6^ irradiated 3T3 feeder cells were grown in CS-FCS containing RM^+^ medium without PR per well in a 12 well plate in triplicates for different time point for each gene. ATRA was added at 1 µM for 24 h prior to adding AD for up to 4 h after which the 3T3 feeder was removed by squirting PBS/EDTA and qPCR lysates were collected for (**B**) *KRT1*, (**C**) *KRT1*0 and (**D**) *KRT2* gene expression analyses. DMSO/EtOH was used as vehicle control (0.001%/0.01% mixture) for ATRA treated samples. All data are shown as relative expression normalised to DMSO/EtOH control and two reference genes, *POLR2A* and *YAP1*. Statistical analyses: n = 3, Error bars =SEM. Two-way ANOVA (Time, Treatment) was used to calculate p values in (**B**–**D**), one-way ANOVA in (**A**), p values are given by asterisks (ns = p > 0.05, *p < 0.05, **p < 0.01 and ****p < 0.0001).

As the steady state level of *KRT2* mRNA was responding differently to ATRA compared with *KRT1* and *KRT10*, we decided to investigate their relative decay rate by transiently blocking mRNA synthesis using actinomycin D (AD). Initial experiments suggested that 2 µg/ml AD was sufficient to inhibit transcription of *KRT1*, *KRT10*, *KRT2* and *c-MYC* genes in NHEK. We used *c-MYC* as a control since its mRNA is reported to have a short half-life^54^. NHEK with feeder cells were grown in SLP^−^/PR^−^ RM^+^ for 24 h and treated with 2 µg/ml AD for up to 4 h and at different time intervals cells were lysed for qPCR analysis. The mRNA expression for *KRT1*, *KRT10*, *KRT2* and *c-MYC* normalised for *POLR2A* and *YAP1* reference genes is shown in Fig. 7A. Both *KRT1* and *KRT10* mRNA were very stable (half-life of more than 4 h) whereas *KRT2* and *c-MYC* had a half-life of about 1 h and reached almost undetectable levels at 4 h (Fig. 7A). This showed that *KRT2* mRNA in NHEK cells had lower half-life compared with *KRT1* and *KRT10* mRNA.

### Effect of ATRA on *KRT1*, *KRT10* and *KRT2* mRNA stability in NHEK

To study the effect of ATRA on the rate of *KRT1*, *KRT1*0 and *KRT2* mRNA decay, we grew NHEK with feeder cells in SLP^−^/PR^−^ RM^+^ for 24 h with or without 1 µM ATRA. AD at 2 µg/ml was used to inhibit transcription and samples were lysed at different time for qPCR analyses. Addition of AD to ATRA treated NHEK showed faster decay rate with half-life of ~15 min for *KRT1* and ~45 min for *KRT10* mRNA compared with *KRT2* which was significantly stabilised with increase in half-life from ~1 h to ~3.5 h. These observations suggest that ATRA treatment of NHEK accelerated the decay of *KRT1* and *KRT10* mRNA but had the opposite effect on *KRT2* mRNA (Fig. 7B–D).

## Discussion

Although FCS and PR are routinely found in medium used to culture epidermal keratinocytes, the effect of SLP, present in FCS, and PR themselves on keratinocyte differentiation have never been fully investigated. In the literature, researchers have identified the potential for influence and used de-lipidised serum^34,35^, CS-FCS^55,56^ or serum free medium^39,57^ supplemented with an effector (either retinoid or steroid) to investigate its specific effect on differentiation. In some studies, low serum concentration in the medium has been used to dilute out any effect of serum lipids^43,58^. Despite their ubiquitous presence in culture media no study has systematically compared keratin expression in medium containing FCS with CS-FCS or studied the effect of PR on the expression of differentiation-specific keratins. In this study, we have compared the expression of differentiation-specific keratin genes *KRT1*, *KRT10* and *KRT2* in FCS with CS-FCS with or without PR and showed how SLP and PR substantially influence differentiation-specific keratin expression.

We compared the effect of SLP and PR on the expression of differentiation-specific keratins in NHEK with two cell lines, HaCaT and N/TERT-1, widely used as substitutes for normal keratinocytes^48–51^. Removal of SLP and PR from the medium increased mRNA and protein expression for *KRT1*, *KRT10* and *KRT2* genes in NHEK (Figs. 1 and 2). When primary keratinocytes are isolated from skin, K2 shows strong expression on western blotting^20^ however it is rapidly downregulated and becomes undetectable when primary epidermal keratinocytes are cultured^19^, therefore, it was surprising to see K2 induction by removing SLP and PR from the medium. Although the induction of K2 was very low, nevertheless it was detectable (Fig. 1E). This is the first study where detectable quantity of K2 has been induced in 2D cultures of NHEK and it is possible that the quantity could be improved by further manipulation of culture conditions. Neither HaCaT nor N/TERT-1 expressed K2 under any experimental condition used in this study. Although HaCaT in 3D cultures do form stratified layers they do not form stratum corneum and do not express precursors of cornified envelops such as filaggrin, loricrin and involucrin as much as in NHEK^49,59^ so it was not surprising they did not express K2. Expression patterns of *KRT1* and *KRT10* transcripts in HaCaT in the absence of SLP and PR were similar to NHEK (compare Fig. 1A,B with Fig. 3A,B). Interestingly, expression of *KRT2* mRNA in HaCaT was significantly increased in the presence of SLP when there was no PR which was very different for NHEK (compare Figs. 1C with 3C). N/TERT-1, on the other hand, has been shown to have normal differentiation characteristics in monolayer and 3D organotypic cultures with proper stratum corneum formation similar to that observed with NHEK^50,60^. Surprisingly, removal of SLP did not induce either *KRT1*, *KRT10* or *KRT2* expression in N/TERT-1 cells. However, the transcripts of all the three keratins showed increased expression in the presence of SLP without PR which was also reflected in protein expression (Fig. 4A–D). This indicates that PR suppresses differentiation-specific expression of keratins in N/TERT-1 cells but only in the presence of SLP. This effect of SLP and PR was very different to that observed in NHEK and HaCaT cells and perhaps reflects the method used to immortalise N/TERT-1 cells.

FCS is an ill-defined component of most culture media, which contains a wide range of biological molecules including growth factors, hormones, vitamins, serum proteins, fatty acids, lipids including retinoids and minerals that are needed by the cells for their growth^61^. Removing the lipids from the serum by charcoal stripping would deplete the serum of retinoids (retinol + ATRA) and that could influence the keratin gene expression observed in this study. Although using 1 µM ATRA in CS-FCS containing medium suppressed expression of at least *KRT1* and *KRT10* transcripts, it is far higher than the physiological levels of retinoids found in FCS and therefore could not be the sole reason for the change in keratin gene expression. The level of retinoids (retinol + ATRA) in bovine serum reported in the literature is between 600–700 nM^62^ and therefore our culture media containing 10% FCS should contain between 60–70 nM of retinoids. Removal of this quantity of retinoids is unlikely to affect keratin gene expression as much as shown in Figs. 1–4. This suggests that charcoal treatment is perhaps removing additional components from CS-FCS which might be keeping keratin expression suppressed. A recent study by Tu and co-workers used proteomic analysis to show that FCS contains a total of 143 proteins out of which 14 including insulin-like growth factor 2 (IGF-2), IGF binding protein (IGFBP)-2 and -6 were reduced by charcoal treatment^56^. Presence of these 14 proteins in FCS could be suppressing the expression of *KRT1*, *KRT10* and *KRT2*. Reduced level of these proteins in CS-FCS by charcoal could be responsible for inducing the gene expression as shown in Figs. 1–4.

The expression of both *KRT1* and *KRT10* was suppressed when 1 µM ATRA was added in RM^+^ containing CS-FCS (Fig. 5). However, presence of PR appears to reduce the effect of ATRA on *KRT1*, but not on *KRT10* expression (compare PR^−^ with PR^+^ in Fig. 5A,B), which was very similar to the effect of SLP (compare SLP^+^/PR^−^ and SLP^+^/PR^+^ in Fig. 1A,B). The effect of ATRA on *KRT2* mRNA was the most pronounced in the absence of PR increasing by more than 2 folds, which is consistent with DNA microarray data showing increase in *KRT2* expression in retinoid exposed keratinocytes^39^. In the presence of PR however there was no increase in *KRT2* expression (Fig. 5C). This effect of PR on keratin expression was novel, it was investigated further by adding back PR in PR-free medium. Both ATRA and PR on their own suppressed expression of *KRT1* and *KRT10* but the effect was not synergistic and when added together PR reduced the effect of ATRA (Fig. 6). Unlike *KRT1* and *KRT10*, the *KRT2* expression either increased (Fig. 5C) or did not change (Fig. 6C) by ATRA suggesting *KRT2* behaved differently. Taken together, these results suggest that the effect of SLP on keratin expression could be primarily due to the bioactivity of retinoids present in FCS.

It has been proposed that due to structural similarity between PR and oestrogen, PR mimics the activity of oestrogen and at concentrations used in tissue culture media, it can increase cell proliferation by 200% and progesterone receptor content of MCF-7 cells by 300%^52,53^. However, later studies have suggested that the oestrogenic activity of PR was primarily because of lipophilic impurities contaminating the commercial preparations of PR^63,64^. This was also shown by our data as PR was able to suppress *KRT1*, *KRT10* and KRT2 expression however ED did not suppress keratin expression, instead stimulating the expression only at certain concentrations suggesting that PR works through a different mechanism from ED (Fig. 6; Supplementary Fig. S4).

The mRNA abundance for a protein is determined by the balance between rates of gene transcription and mRNA half-life^65^. Our measurement of transient mRNA decay rates showed significant differences in *KRT1*, *KRT10* and *KRT2* mRNA in RM^+^ medium containing CS-FCS. This is the first report where mRNA decay rates of differentiation-specific keratins have been investigated. Surprisingly, *KRT2* mRNA had the fastest decay rate compared with *KRT1* and *KRT10* mRNA which did not show significant decay over 4 h (Fig. 7A). These results suggest that the rapid mRNA decay together with the transcriptional suppression could be factors which makes K2 protein mostly undetectable in 2D keratinocyte cultures^19^. The mRNA decay rates are regulated by *cis*-acting sequence determinants, which recruit *trans-*acting factors and largely control mRNA stability. One of the most characterised *cis*-elements regulating mRNA decay are AU-rich elements (ARE) located in the 3′ untranslated region (UTR) of 5–8% of human genes of short-lived mRNA which function as a signal for rapid degradation^66,67^. There are three classes of ARE, class I contains multiple, overlapping copies of AUUUA motifs dispersed over the 3′ UTR surrounded by U-rich regions; class II contains multiple, overlapping copies of AUUUA, and class III lacks AUUUA but contains predominantly U-rich sequences^68^. These ARE recruit AU-binding proteins which assemble the degradation machinery to mRNA for their destruction^68,69^. ARE mediated decay cannot be the mechanism for the regulation of *KRT1* and *KRT10* expression because of two reasons, first their mRNA were stable with no significant change over 4 h suggesting the half-life must be more than 4 h (Fig. 7A) and second, the 3′ UTR of none of them contained any class of ARE. The *KRT2* mRNA was however less stable but as it also did not contain a typical ARE so should be degraded involving mechanisms other than ARE. In addition to 3′ ARE, other regions have been identified in the coding sequence of a number of genes including *c-FOS*^70^, *c-MYC*^71^ and *β*-tubulin^65,72^, which determine stability of individual transcript and it is possible that *KRT2* mRNA stability is also regulated by sequences in its coding region.

Although retinoids suppress transcription of most keratin genes by direct interaction between ligand bound receptors and retinoid responsive elements on keratin genes^44,45^, the steady-state mRNA for several keratins including *KRT2*, *KRT4*, *KRT7*, *KRT13*, *KRT15* and *KRT19* have been shown to increase^39,40,43,58,73^. In this study we show that steady state mRNA for *KRT1* and *KRT10* is reduced by ATRA whereas for *KRT2* it is induced. One possible explanation could be that the transcription of *KRT1/KRT10* and *KRT2* are reciprocally regulated by ATRA. This is supported by the fact that normal expression of K1 and K2 is mutually exclusive at different body sites of the murine skin^74^. Another possible mechanism could be the ATRA induced stabilisation of *KRT2* transcript as has been proposed for *KRT19* for the increase in its steady-state mRNA^43^. In the presence of ATRA the *KRT1* transcripts become most unstable (half-life reduced from more than 4 h to just 15 min), followed by *KRT10* (half-life reduced from more than 4 h to 45 min) whilst *KRT2* mRNA becomes more stable (half-life increased from 1 h to 3.5 h). It has previously been reported that exposure of cells to retinoic acid also stabilises transcripts for a number of genes including proteolipid protein^75^, prolactin^76^, *KRT19*^43^ and calbindin_D28K^77^. At the same time destabilisation of transcripts for TNF-α^78^ and fibroblast growth factor^79^ by retinoic acid has also been reported. Although the exact mechanism of transcript stabilisation or destabilisation by retinoic acid is not clear, a number of proteins have been identified which bind to transcripts to regulate stability (reviewed in^80^). One of the best characterised is HuR, also known as embryonic lethal abnormal visual-like protein 1 (ELAVL1), family of proteins which are ubiquitously expressed^81^. These proteins strongly bind specific transcripts and protect them from the degradation machinery. When cells are exposed to ATRA cellular retinoic acid binding proteins I and II (CRABPs) are activated in the cytoplasm. It is tempting to speculate that ATRA-CRABP complexes could dissociate the transcript-protecting proteins thereby inducing transcript degradation as has been observed for *K1* and *K10* (Fig. 7A–C). It is also conceivable that the ATRA-CRABP complex could attract other proteins to a specific transcript thereby increasing their stability as has been observed for *KRT2* mRNA.

In summary, we have shown that CS-FCS induced expression of differentiation-specific keratins *KRT1*, *KRT10* and *KRT2* in NHEK. The HaCaT and N/TERT-1, the two cell lines frequently used as a substitute for NHEK, the effect of SLP and PR was significantly different from NHEK. The effect of SLP was mimicked by ATRA which suppressed the expression of *KRT1* and *KRT10* but increased *KRT2* expression. PR on its own also suppressed expression of these keratin genes. We observed that the increase in *KRT2* transcript was because of stabilisation of its transcript by ATRA whereas *KRT1* and *KRT10* mRNA were destabilised. This suggests that post-transcriptional mechanism plays an important role in retinoid mediated regulation of differentiation-specific keratins. Overall this study emphasises the need to better understand how *in vitro* culture conditions affect the differentiation mechanism of keratinocytes, which could otherwise result in misinterpretation of biomarkers for skin and mucosal pathologies characterised by dysregulated keratinocyte differentiation such as cancer, inflammatory diseases or wound healing.

## Methods and Materials

### Cell lines and antibodies

NHEK derived from a pool of a minimum of three neonatal foreskins (Gibco, Invitrogen) were cultured in RM^+^/feeder system^82,83^. N/TERT-1 keratinocytes, immortalised by overexpression of hTert and downregulation of p16^60^, and HaCaT, a spontaneously immortalised cell line developed from human adult skin^59^, were grown in RM^+^ medium. Normal dermal fibroblasts, provided by Dr. Amir Sharili (QMUL, London), were cultured in DMEM + 10% FCS. Murine 3T3 fibroblasts, provided by Professor Kenneth Parkinson (QMUL, London), to be used as feeder for co-culturing NHEK were grown in DMEM with 10% donor bovine serum. The 3T3s were irradiated using 60 Gy of γ radiation to stop their proliferation irreversibly. These cells were used to support the growth of NHEK in 2D and 3D organotypic cultures. The antibodies against K1 (ab81623), K2 (ab19122), K10 (ab9025) and GAPDH (ab9485) were obtained from Abcam UK. Anti-K14 (clone LLOO1) was available as culture supernatant in our laboratory^18^. Peroxidase conjugated anti-mouse IgG (AP124P) and anti-rabbit IgG (AP132P) both produced in goat were purchased from Millipore, UK. Peroxidase conjugated anti-mouse IgG (NA931) produced in sheep was bought from GE Healthcare. Alexa Fluor 488 labelled goat anti-mouse IgG (H + L) (A-11029) and goat anti-rabbit F(ab’)2 IgG (H + L) (A-11070) were purchased from Life Technologies, UK.

### Culture medium

RM^+^ (Rheinwald-Green Modified, also called FAD) medium^82,83^ was prepared as described previously^84^. Cells were cultured in four different formulations of RM^+^ medium; with (SLP^−^) or without (SLP^+^) charcoal treatment and with (PR^+^) or without (PR^−^) PR.

### 3D Organotypic cultures

NHEK cells used in 3D organotypic (OT) cultures were cultured in RM^+^ with appropriate formulation prior to setting the 3D system. To set up OT, plastic inserts (pore size 0.4 µm) were placed in 12-well plates. Rat tail collagen solution was prepared on ice at 4 mg/ml in DMEM either with or without PR and neutralised using 0.5 N NaOH. Primary dermal fibroblasts were used in collagen matrix to support the growth of keratinocytes. Next day, medium was aspirated from inside and outside of each insert and 1 ml of RM^+^ (with or without PR) and (with normal FCS or CS-FCS) was added underneath the inserts. The cells were allowed to grow at an air-liquid interface for 10 days to induce stratification and the medium underneath the inserts was changed every day^85^.

### Cell treatment

NHEK were co-cultured in 6 or 12 well plates with γ-irradiated 3T3 murine fibroblasts as feeder to support keratinocyte growth in different RM^+^ formulations for 3 days before removing the feeder and using keratinocytes for gene expression analysis by qPCR (cells grown in 12 well plates) and western blotting (cells grown in 6 well plates). For specific treatment, the co-cultures were seeded in 12 well plate, grown in RM^+^ containing CS-FCS, next day the cells were treated with reagents, ATRA (R2625, Sigma) or ED (E8875, Sigma) for 24 h. ATRA was first dissolved in DMSO and further diluted in ethanol (EtOH) to the desired concentrations. The concentrations of DMSO/EtOH used as a vehicle control varied from 0.003% to 0.03% (v/v). ED was dissolved in DMSO and further diluted in culture medium to working dilutions. DMSO (0.001%) was used as a vehicle control. PR (P3532, Sigma, UK) was added in PR free RM^+^ culture medium at 0.01 mg/ml. The feeders were removed by squirting the culture with PBS/EDTA and collecting cell lysates for expression analysis by qPCR. When PR was added, PR free medium was used to grow cells as control. Actinomycin D (AD, 11805-017, Gibco, UK) was diluted from a stock (2 mg/ml in water) to working dilutions.

### mRNA isolation and cDNA synthesis

Cells were seeded at desired density in 6-well or 12-well plates, washed with PBS and lysed after specific treatment or media incubation. Lysates were either stored at −80 °C or subjected to mRNA extraction. Dynabeads mRNA DIRECT kit was used to extract polyadenylated (polyA) mRNA and the total RNA was isolated using RNeasy kit (Qiagen) according to manufacturer’s instructions. The concentration of mRNA was measured using a NanoDrop spectrophotometer. For reverse transcription qPCRBIO cDNA Synthesis Kit (PCR Biosystem, UK) was used according to manufacturer’s instructions. The reverse transcription reaction protocol was as follows: 42 °C for 30 min, 85 °C for 5 min and 4 °C for 5 min. The resulting cDNA was diluted using nuclease-free qPCR H_2_O and immediately used in qPCR gene expression analysis or stored at −20 °C for later use.

### Real-time quantitative PCR (qPCR)

Five micromolar forward (F) and reverse (R) primer mixes were made by mixing equal volumes of forward and reverse primer stocks (100 µM) with suitable amount of nuclease-free H_2_O. The qPCR primer sequences used for different genes in this study along with amplicon size in parenthesis were as follows. *KRT1* (128 bp): F (CGGAACTGAAGAACATGCAG), R (CATATAAGCACCATCCACATCC); *KRT10* (134 bp): F (AAACCATCGATGACCTTAAAAATC), R (GCGCAGAGCTACCTCATTCT); *KRT2* (95 bp): F (GCCTCCTTCATTGACAAGGT), R (CGGGTGCCAACATTCATT); *c-MYC* (138 bp): F (CACCAGCAGCGACTCTGA), R (CTGTGAGGAGGTTTGCTGT; *POLR2A* (128 bp): F (AGGAGTTTCGGCTCAGTGG), R (AGGTTCTCCAAGGGACTGC); *YAP1* (128 bp): F (ACTGCTTCGGCAGGTGAG), R (TCGTCATTGTTCTCAATTCCTG). For PCR amplification 384-well format plates were used in a total volume of 5 μl per well consisting of 2.5 µl qPCRBIO SyGreen Blue Mix Lo Rox (#PB20.11-50, PCRBIO Systems, UK), 0.5 µl 5 µM F/R primer mix and 2 µl cDNA template. The cDNA samples were loaded at the bottom of the wells, followed by a mixture of SYBR Green and F/R primer added at the top wall of each well. Plates were sealed, centrifuged and inserted into the LightCycler 480 machine (Blizard institute’s core facility) for qPCR according to the recommended protocol. Relative quantification of mRNA expression was measured using LightCycler 480 software (release 1.5.0). For normalisation, *POLR2A* and *YAP1* were used as reference genes^51^.

### Immunostaining of cells grown in 2 and 3D cultures

NHEK cells were trypsinised, counted and grown under different growth conditions on the top of collagen coated glass coverslips with irradiated 3T3 feeder cells. After 24 h, the cells were fixed with a mixture of acetone and methanol (1:1) and immunostained for K1, K2, K10 and K14 using the method described previously^84^. Stained samples were imaged using either Leica Epi DM5000 microscope or Leica Epi DM4000 equipped with a DFC350 FX digital camera under 20×/0.5 NA and/or 40×/0.75 NA objective lenses. The images were acquired by Metamorph imaging system and processed using ImageJ bundled with 64-bit Java 1.8.0_112 (freely available at https://imagej.nih.gov/ij/download.html).

The 3D reconstructed plugs were fixed in 4% (w/v) paraformaldehyde in PBS, dehydrated, paraffin embedded and cut into 5 µm thick sections. The sections were de-waxed, re-hydrated, H & E stained, de-hydrated and mounted using DPX medium. Nikon Eclipse 80i Stereology Microscope was used for imaging. To immunostain, the sections of 3D cultures were first antigens retrieved, followed by immunostaining with antibodies against K1, K2, K10 and K14 as described recently^84^.

### Protein isolation and western blotting

Keratinocytes were seeded in 6-well plates with the required media for each experiment, lysed in 200 µl/well lysis buffer [4% (w/v) SDS, 20% (v/v) glycerol, 125 mM Tris-HCl, pH 6.8] on ice. Cell lysates were collected, sonicated 3 ×5 seconds, heated (90–100 °C) for 5 min, spun at 15000 rpm for 10 min to remove cell debris and stored at −80 °C for later use. Protein concentration was determined using DC Protein Assay Kit (Bio-Rad) using BSA as standard. 2-mercaptoethanol 10% (v/v) and Bromophenol blue (0.1% (w/v) were also added to the protein lysates and loaded onto a pre-cast 4–12% (w/v) polyacrylamide NuPage gels and the proteins were separated electrophoretically using NuPage MES SDS running buffer. The separated proteins were transferred onto nitrocellulose membranes, probed first with primary antibodies and then peroxidase-conjugated, secondary antibodies as described previously^84^. After washing, the membranes were developed using ECL Prime Detection Reagent according to the manufacturers’ instructions. The membranes were then imaged using ChemiDoc imager (Blizard institute’s core facility) and Images were analysed using ImageJ or Image Lab v5.1 (freely available at https://www.bio-rad.com/en-uk/product/image-lab-software?ID = KRE6P5E8Z).

### Statistical analysis

All experiments were performed for a minimum in triplicates. Student’s t-test were performed using Microsoft Excel and all results were presented as the mean of 3 individual experiments with standard error of the mean (S.E.M). The p values were calculated and were considered significant if below 0.05. One-way (1 variable) or two-way (2 variables) analysis of variance (ANOVA) was also performed using Microsoft Excel data analysis tool. ANOVA was used to test the null hypothesis of significant difference if p values were lower than 0.05.

## Supplementary information

Supplementary information.

## Data Availability

All data generated or analysed during this study are included in this manuscript.

## References

[1] Lee SH, Jeong SK, Ahn SK (2006). An update of the defensive barrier function of skin. Yonsei Med. J..

[2] Madison KC (2003). Barrier function of the skin: “la raison d’etre” of the epidermis. J. Invest. Dermatol..

[3] Park K (2015). Role of micronutrients in skin health and function. Biomol. Ther..

[4] Morris RJ, Potten CS (1994). Slowly cycling (label-retaining) epidermal cells behave like clonogenic stem cells *in vitro*. Cell Prolif..

[5] Blanpain C, Fuchs E (2006). Epidermal stem cells of the skin. Annu. Rev. Cell Dev. Biol..

[6] Wikramanayake TC, Stojadinovic O, Tomic-Canic M (2014). Epidermal Differentiation in Barrier Maintenance and Wound Healing. Adv. Wound Care.

[7] Ojeh N, Pastar I, Tomic-Canic M, Stojadinovic O (2015). Stem Cells in Skin Regeneration, Wound Healing, and Their Clinical Applications. Int. J. Mol. Sci..

[8] Bragulla HH, Homberger DG (2009). Structure and functions of keratin proteins in simple, stratified, keratinized and cornified epithelia. J. Anat..

[9] Hatzfeld M, Franke WW (1985). Pair formation and promiscuity of cytokeratins: formation *in vitro* of heterotypic complexes and intermediate-sized filaments by homologous and heterologous recombinations of purified polypeptides. J. Cell Biol..

[10] Hatzfeld M, Weber K (1990). The coiled coil of *in vitro* assembled keratin filaments is a heterodimer of type I and II keratins: use of site-specific mutagenesis and recombinant protein expression. J. Cell Biol..

[11] Moll R, Franke WW, Schiller DL, Geiger B, Krepler R (1982). The catalog of human cytokeratins: patterns of expression in normal epithelia, tumors and cultured cells. Cell.

[12] Sun TT, Eichner R, Nelson WG, Vidrich A, Woodcock-Mitchell J (1983). Keratin expression during normal epidermal differentiation. Curr. Probl. Dermatol..

[13] Byrne C, Tainsky M, Fuchs E (1994). Programming gene expression in developing epidermis. Dev..

[14] Lloyd C (1995). The basal keratin network of stratified squamous epithelia: defining K15 function in the absence of K14. J. Cell Biol..

[15] Waseem A (1999). Keratin 15 expression in stratified epithelia: downregulation in activated keratinocytes. J. Invest. Dermatol..

[16] Fuchs E, Green H (1980). Changes in keratin gene expression during terminal differentiation of the keratinocyte. Cell.

[17] Stoler A, Kopan R, Duvic M, Fuchs E (1988). Use of monospecific antisera and cRNA probes to localize the major changes in keratin expression during normal and abnormal epidermal differentiation. J. Cell Biol..

[18] Purkis PE (1990). Antibody markers of basal cells in complex epithelia. J. Cell Sci..

[19] Collin C, Moll R, Kubicka S, Ouhayoun JP, Franke WW (1992). Characterization of human cytokeratin 2, an epidermal cytoskeletal protein synthesized late during differentiation. Exp. Cell Res..

[20] Bloor BK (2003). Expression of keratin K2e in cutaneous and oral lesions: association with keratinocyte activation, proliferation, and keratinization. Am. J. Pathol..

[21] Leigh IM (1995). Keratins (K16 and K17) as markers of keratinocyte hyperproliferation in psoriasis *in vivo* and *in vitro*. Br. J. Dermatol..

[22] Machesney M, Tidman N, Waseem A, Kirby L, Leigh I (1998). Activated keratinocytes in the epidermis of hypertrophic scars. Am. J. Pathol..

[23] Moll R, Divo M, Langbein L (2008). The human keratins: biology and pathology. Histochem. Cell Biol..

[24] Banks-Schlegel S, Green H (1981). Involucrin synthesis and tissue assembly by keratinocytes in natural and cultured human epithelia. J. Cell Biol..

[25] Crish JF, Howard JM, Zaim TM, Murthy S, Eckert RL (1993). Tissue-specific and differentiation-appropriate expression of the human involucrin gene in transgenic mice: an abnormal epidermal phenotype. Differ..

[26] Presland RB (1997). Evidence for specific proteolytic cleavage of the N-terminal domain of human profilaggrin during epidermal differentiation. J. Invest. Dermatol..

[27] Ishida-Yamamoto A, Hohl D, Roop DR, Iizuka H, Eady RA (1993). Loricrin immunoreactivity in human skin: localization to specific granules (L-granules) in acrosyringia. Arch. Dermatol. Res..

[28] Mehrel T (1990). Identification of a major keratinocyte cell envelope protein, loricrin. Cell.

[29] Fujimoto W (1993). Expression of cornifin in squamous differentiating epithelial tissues, including psoriatic and retinoic acid-treated skin. J. Invest. Dermatol..

[30] Eckert RL, Sturniolo MT, Broome AM, Ruse M, Rorke EA (2005). Transglutaminase function in epidermis. J. Invest. Dermatol..

[31] Fell HB, Mellanby E (1953). Metaplasia produced in cultures of chick ectoderm by high vitamin A. J. Physiol..

[32] Randolph, R. K. & Siegenthaler, G. In Retinoids Vol. 139 Handbook of Experimental Pharmacology (eds. Nau, H. & Blaner, W. S.) Ch. Chapter 17, 491–520 (Springer, 1999).

[33] Torma H (2011). Regulation of keratin expression by retinoids. Dermatoendocrinol.

[34] Fuchs E, Green H (1981). Regulation of terminal differentiation of cultured human keratinocytes by vitamin A. Cell.

[35] Green H, Watt FM (1982). Regulation by vitamin A of envelope cross-linking in cultured keratinocytes derived from different human epithelia. Mol. Cell Biol..

[36] Brown LJ, Geesin JC, Rothnagel JA, Roop DR, Gordon JS (1994). Retinoic acid suppression of loricrin expression in reconstituted human skin cultured at the liquid-air interface. J. Invest. Dermatol..

[37] Marvin KW (1992). Cornifin, a cross-linked envelope precursor in keratinocytes that is down-regulated by retinoids. Proc. Natl Acad. Sci. USA.

[38] Sizemore N (1993). Retinoid regulation of human ectocervical epithelial cell transglutaminase activity and keratin gene expression. Differ..

[39] Lee DD (2009). Retinoid-responsive transcriptional changes in epidermal keratinocytes. J. Cell Physiol..

[40] Korge B, Stadler R, Mischke D (1990). Effect of retinoids on hyperproliferation-associated keratins K6 and K16 in cultured human keratinocytes: a quantitative analysis. J. Invest. Dermatol..

[41] Gilfix BM, Eckert RL (1985). Coordinate control by vitamin A of keratin gene expression in human keratinocytes. J. Biol. Chem..

[42] Stellmach V, Leask A, Fuchs E (1991). Retinoid-mediated transcriptional regulation of keratin genes in human epidermal and squamous cell carcinoma cells. Proc. Natl Acad. Sci. USA.

[43] Crowe DL (1993). Retinoic acid mediates post-transcriptional regulation of keratin 19 mRNA levels. J. Cell Sci..

[44] Blumenberg M, Connolly DM, Freedberg IM (1992). Regulation of keratin gene expression: the role of the nuclear receptors for retinoic acid, thyroid hormone, and vitamin D3. J. Invest. Dermatol..

[45] Tomic-Canic M, Day D, Samuels HH, Freedberg IM, Blumenberg M (1996). Novel regulation of keratin gene expression by thyroid hormone and retinoid receptors. J. Biol. Chem..

[46] Maguire A, Morrissey B, Walsh JE, Lyng FM (2011). Medium-mediated effects increase cell killing in a human keratinocyte cell line exposed to solar-simulated radiation. Int. J. Radiat. Biol..

[47] Ehrlich F (2019). Differential Evolution of the Epidermal Keratin Cytoskeleton in Terrestrial and Aquatic Mammals. Mol. Biol. Evol..

[48] Colombo I (2017). HaCaT Cells as a Reliable *In Vitro* Differentiation Model to Dissect the Inflammatory/Repair Response of Human Keratinocytes. Mediators Inflamm..

[49] Schoop VM, Mirancea N, Fusenig NE (1999). Epidermal organization and differentiation of HaCaT keratinocytes in organotypic coculture with human dermal fibroblasts. J. Invest. Dermatol..

[50] Smits JPH (2017). Immortalized N/TERT keratinocytes as an alternative cell source in 3D human epidermal models. Sci. Rep..

[51] Bose A (2012). Two mechanisms regulate keratin K15 expression in keratinocytes: role of PKC/AP-1 and FOXM1 mediated signalling. PLoS One.

[52] Berthois Y, Katzenellenbogen JA, Katzenellenbogen BS (1986). Phenol red in tissue culture media is a weak estrogen: implications concerning the study of estrogen-responsive cells in culture. Proc. Natl Acad. Sci. USA.

[53] Welshons WV, Wolf MF, Murphy CS, Jordan VC (1988). Estrogenic activity of phenol red. Mol. Cell Endocrinol..

[54] Dani C (1984). Extreme instability of myc mRNA in normal and transformed human cells. Proc. Natl Acad. Sci. USA.

[55] Cao Z (2009). Effects of resin or charcoal treatment on fetal bovine serum and bovine calf serum. Endocr. Res..

[56] Tu C (2018). Proteomic Analysis of Charcoal-Stripped Fetal Bovine Serum Reveals Changes in the Insulin-like Growth Factor Signaling Pathway. J. Proteome Res..

[57] Vollberg TM (1992). Retinoic acid receptors as regulators of human epidermal keratinocyte differentiation. Mol. Endocrinol..

[58] Virtanen M, Sirsjo A, Vahlquist A, Torma H (2010). Keratins 2 and 4/13 in reconstituted human skin are reciprocally regulated by retinoids binding to nuclear receptor RARalpha. Exp. Dermatol..

[59] Boukamp P (1988). Normal keratinization in a spontaneously immortalized aneuploid human keratinocyte cell line. J. Cell Biol..

[60] Dickson MA (2000). Human keratinocytes that express hTERT and also bypass a p16(INK4a)-enforced mechanism that limits life span become immortal yet retain normal growth and differentiation characteristics. Mol. Cell Biol..

[61] Gstraunthaler G (2003). Alternatives to the use of fetal bovine serum: serum-free cell culture. ALTEX.

[62] Hagen JJ, Washco KA, Monnig CA (1996). Determination of retinoids by reversed-phase capillary liquid chromatography with amperometric electrochemical detection. J. Chromatogr. B Biomed. Appl..

[63] Bindal RD, Carlson KE, Katzenellenbogen BS, Katzenellenbogen JA (1988). Lipophilic impurities, not phenolsulfonphthalein, account for the estrogenic activity in commercial preparations of phenol red. J. Steroid Biochem..

[64] Bindal RD, Katzenellenbogen JA (1988). Bis(4-hydroxyphenyl)[2-(phenoxysulfonyl)phenyl]methane: isolation and structure elucidation of a novel estrogen from commercial preparations of phenol red (phenolsulfonphthalein). J. Med. Chem..

[65] Ross J (1995). mRNA stability in mammalian cells. Microbiol. Rev..

[66] Shaw G, Kamen R (1986). A conserved AU sequence from the 3′ untranslated region of GM-CSF mRNA mediates selective mRNA degradation. Cell.

[67] Bakheet T, Williams BR, Khabar KS (2006). ARED 3.0: the large and diverse AU-rich transcriptome. Nucleic Acids Res..

[68] Chen CY, Shyu AB (1995). AU-rich elements: characterization and importance in mRNA degradation. Trends Biochem. Sci..

[69] Brewer G, An A (1991). U-rich element RNA-binding factor regulates c-myc mRNA stability *in vitro*. Mol. Cell Biol..

[70] Schiavi SC (1994). Multiple elements in the c-fos protein-coding region facilitate mRNA deadenylation and decay by a mechanism coupled to translation. J. Biol. Chem..

[71] Wisdom R, Lee W (1991). The protein-coding region of c-myc mRNA contains a sequence that specifies rapid mRNA turnover and induction by protein synthesis inhibitors. Genes. Dev..

[72] Yen TJ, Machlin PS, Cleveland DW (1988). Autoregulated instability of beta-tubulin mRNAs by recognition of the nascent amino terminus of beta-tubulin. Nat..

[73] Virtanen M, Torma H, Vahlquist A (2000). Keratin 4 upregulation by retinoic acid *in vivo*: a sensitive marker for retinoid bioactivity in human epidermis. J. Invest. Dermatol..

[74] Fischer H (2014). Loss of keratin K2 expression causes aberrant aggregation of K10, hyperkeratosis, and inflammation. J. Invest. Dermatol..

[75] Lopez-Barahona M (1993). Retinoic acid posttranscriptionally up-regulates proteolipid protein gene expression in C6 glioma cells. J. Biol. Chem..

[76] Gellersen B, Kempf R, Hartung S, Bonhoff A, DiMattia GE (1992). Posttranscriptional regulation of the human prolactin gene in IM-9-P3 cells by retinoic acid. Endocrinol..

[77] Wang YZ, Christakos S (1995). Retinoic acid regulates the expression of the calcium binding protein, calbindin-D28K. Mol. Endocrinol..

[78] Motomura K, Ohata M, Satre M, Tsukamoto H (2001). Destabilization of TNF-alpha mRNA by retinoic acid in hepatic macrophages: implications for alcoholic liver disease. Am. J. Physiol. Endocrinol. Metab..

[79] Liaudet-Coopman ED, Wellstein A (1996). Regulation of gene expression of a binding protein for fibroblast growth factors by retinoic acid. J. Biol. Chem..

[80] Staton JM, Thomson AM, Leedman PJ (2000). Hormonal regulation of mRNA stability and RNA-protein interactions in the pituitary. J. Mol. Endocrinol..

[81] Fan XC, Steitz JA (1998). Overexpression of HuR, a nuclear-cytoplasmic shuttling protein, increases the *in vivo* stability of ARE-containing mRNAs. EMBO J..

[82] Rheinwald JG, Green H (1975). Serial cultivation of strains of human epidermal keratinocytes: the formation of keratinizing colonies from single cells. Cell.

[83] Rheinwald JG, Green H (1977). Epidermal growth factor and the multiplication of cultured human epidermal keratinocytes. Nat..

[84] Aldehlawi H (2019). The monoclonal antibody EPR1614Y against the stem cell biomarker keratin K15 lacks specificity and reacts with other keratins. Sci. Rep..

[85] Parenteau NL, Bilbo P, Nolte CJ, Mason VS, Rosenberg M (1992). The organotypic culture of human skin keratinocytes and fibroblasts to achieve form and function. Cytotechnology.

