# Supplementary material for: Serum lipids, retinoic acid and phenol red differentially regulate expression of keratins K1, K10 and K2 in cultured keratinocytes
Source: Sci Rep. 2020 Mar 16;10:4829. doi: 10.1038/s41598-020-61640-9 (PMC7076045; doi:10.1038/s41598-020-61640-9)
Supplement: Supplementary file 1 — Supplementary information. [file 41598_2020_61640_MOESM1_ESM.pdf]

Serum lipids, retinoic acid and phenol red differentially regulate expression of keratins K1, K10 and K2 in cultured keratinocytes

Hebah Aldehlawi, Saima Usman, Anand Lalli, Fatima Ahmad, Gianne Williams, Muy-Teck Teh, and Ahmad Waseem

<u>Supplementary Information</u>

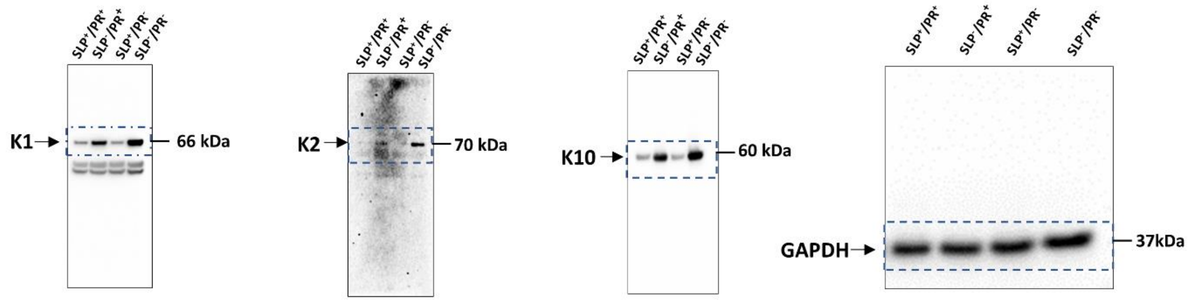

Supplementary figure S1: Raw western blot data corresponding to the cropped blots shown in the figure 1D. Western blotting of keratins extracted from NHEK cells after growing them for 3 days in four different RM+ media conditions (SLP^{+}/PR^{+}, SLP^{-}/PR^{+}, SLP^{+}/PR^{-}, SLP^{-}/PR^{-}) using (A) K1, (B) K10 and (C) K2 antibodies. (D) GAPDH was used as loading control. The cropped area is shown in inset. The lower molecular bands in (A) were non-specific as their intensity did not change in different culture conditions.

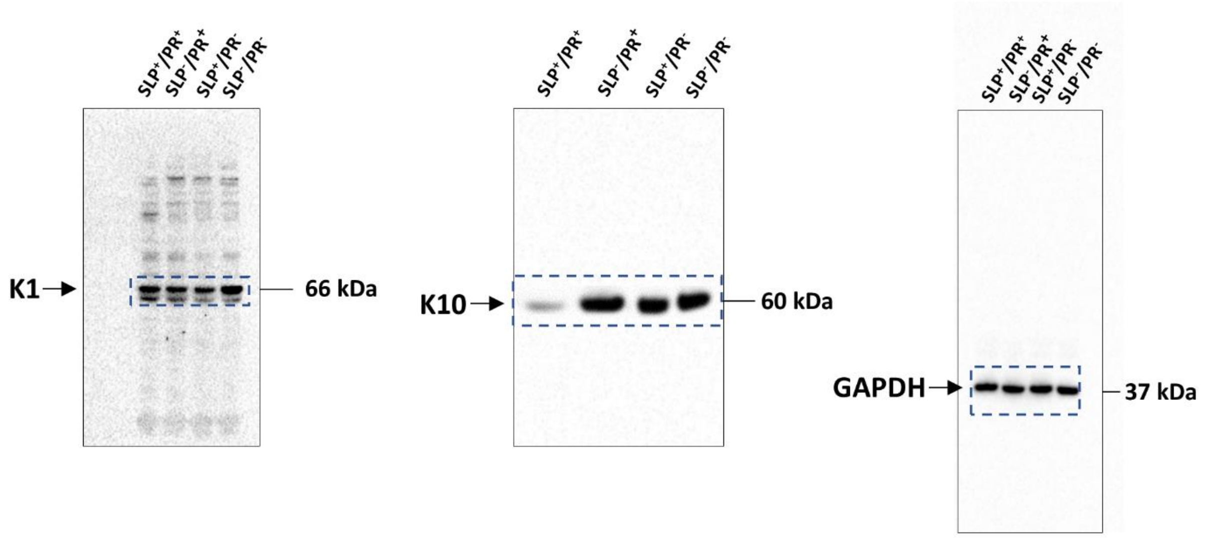

Supplementary figure S2: Raw western blot data corresponding to the cropped blots shown in the figure 3D. Western blotting of keratins extracted from HaCaT cells after growing them in the four different RM+ media conditions (SLP$^+$/PR$^+$, SLP$^-$/PR$^+$, SLP$^+$/PR$^-$, SLP$^-$/PR$^-$) using antibodies specific for (A) K1 and (B) K10. (C) GAPDH was used as loading control. The cropped area is shown in inset.

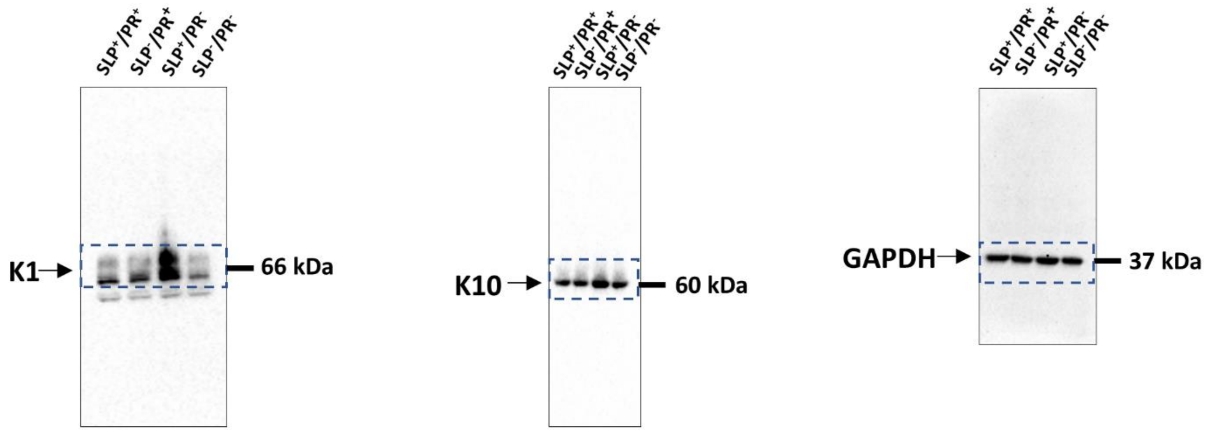

Supplementary figure S3: Raw western blot data corresponding to cropped blots shown in the figure 4D. Western blotting of keratins extracted from N/Tert-1 cells after growing them for 3 days in the four different RM^+ media conditions (SLP^+/PR^+, SLP^-/PR^+, SLP^+/PR^-, SLP^-/PR^-) using (A) K1 and (B) K10 antibodies. (C) GAPDH was used as loading control. The cropped area is shown in inset.

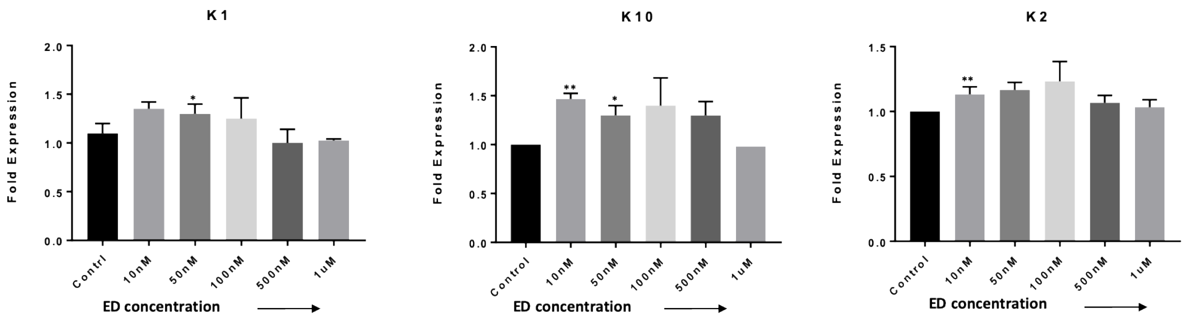

Supplementary figure S4. Effect of ED on the expression of *KRT1*, *KRT10* and *KRT2* genes in NHEK. NHEK cells were grown with irradiated 3T3 feeder cells in CS-FCS and PR free RM^+ medium. ED dissolved in DMSO (0.001% final concentration) was added in the range of 10 nM – 1 µM in the culture medium for 24 h before lysing for *KRT1*, *KRT10* and *KRT2* expression analysis by qPCR. Control cells were treated only with DMSO (0.001%). Data is shown as fold expression normalised to the expression of two housekeeping genes, *POLR2A* and *YAP1*. Statistical analysis: n=3, Error bars=SEM, Student's t-test was performed to calculate p values (*=p<0.05 and **=p<0.01).
